# Effect of Early Life Exposure to Air Pollution on Development of Childhood Asthma

**DOI:** 10.1289/ehp.0900916

**Published:** 2009-10-08

**Authors:** Nina Annika Clark, Paul A. Demers, Catherine J. Karr, Mieke Koehoorn, Cornel Lencar, Lillian Tamburic, Michael Brauer

**Affiliations:** 1 School of Population and Public Health and; 2 School of Environmental Health, University of British Columbia, Vancouver, British Columbia, Canada; 3 Department of Pediatrics, University of Washington, Seattle, Washington, USA; 4 Centre for Health Services and Policy Research, University of British Columbia, Vancouver, British Columbia, Canada

**Keywords:** administrative data, air pollution, asthma, children’s health, in utero, respiratory, traffic

## Abstract

**Background:**

There is increasing recognition of the importance of early environmental exposures in the development of childhood asthma. Outdoor air pollution is a recognized asthma trigger, but it is unclear whether exposure influences incident disease. We investigated the effect of exposure to ambient air pollution *in utero* and during the first year of life on risk of subsequent asthma diagnosis in a population-based nested case–control study.

**Methods:**

We assessed all children born in southwestern British Columbia in 1999 and 2000 (*n* = 37,401) for incidence of asthma diagnosis up to 3–4 years of age using outpatient and hospitalization records. Asthma cases were age- and sex-matched to five randomly chosen controls from the eligible cohort. We estimated each individual’s exposure to ambient air pollution for the gestational period and first year of life using high-resolution pollution surfaces derived from regulatory monitoring data as well as land use regression models adjusted for temporal variation. We used logistic regression analyses to estimate effects of carbon monoxide, nitric oxide, nitrogen dioxide, particulate matter ≤ 10 μm and ≤ 2.5 μm in aerodynamic diameter (PM_10_ and PM_2.5_), ozone, sulfur dioxide, black carbon, woodsmoke, and proximity to roads and point sources on asthma diagnosis.

**Results:**

A total of 3,482 children (9%) were classified as asthma cases. We observed a statistically significantly increased risk of asthma diagnosis with increased early life exposure to CO, NO, NO_2_, PM_10_, SO_2_, and black carbon and proximity to point sources. Traffic-related pollutants were associated with the highest risks: adjusted odds ratio = 1.08 (95% confidence interval, 1.04–1.12) for a 10-μg/m^3^ increase of NO, 1.12 (1.07–1.17) for a 10-μg/m^3^ increase in NO_2_, and 1.10 (1.06–1.13) for a 100-μg/m^3^ increase in CO. These data support the hypothesis that early childhood exposure to air pollutants plays a role in development of asthma.

Asthma is the most common chronic disease in childhood [World Health Organization (WHO) 2006]. Its prevalence is high and has generally increased worldwide over the latter part of the 20th century (Asher et al. 2006; WHO 2006). Although explanations for relatively rapid changes in prevalence are unknown, environmental factors, independently and jointly with genetic factors, are thought to be responsible. Although air pollution has been consistently shown to exacerbate existing asthma (English et al. 1999; Lipsett et al. 1997; McConnell et al. 1999, 2006; Nicolai et al. 2003; Norris et al. 1999), there are few investigations of asthma onset and air pollution despite the hypothesized link with exposure to outdoor air pollution (Institute of Medicine 2000; von Mutius 2000).

Earlier studies have generally relied on simple measures of traffic proximity and density to estimate exposure and have not found an association between air pollution and asthma incidence (Ciccone et al. 1998; English et al. 1999; Wjst et al. 1993). More recent studies have used modeling approaches that provide high-resolution estimates of neighborhood-scale variations in air pollution. Several studies using this approach have observed increases in asthma incidence or asthma symptoms for children exposed to higher levels of traffic-related air pollution (Brauer et al. 2002, 2007; Gauderman et al. 2005; McConnell et al. 2006; Morgenstern et al. 2008; Zmirou et al. 2004). However, not all such studies of this type have reported consistent associations (Gehring et al. 2002; Zmirou et al. 2004).

Pre- and postbirth exposures to environmental tobacco smoke (ETS) are independently associated with increased asthma incidence (Haberg et al. 2007). Although air pollution exposures before 2–3 years of age appear to be most important for asthma development (McConnell et al. 2006; Zmirou et al. 2004), the effect of prebirth (or *in utero*) exposure has not to our knowledge been examined. One exception is a study of polyclyclic aromatic hydrocarbon exposure, which has been examined in conjunction with ETS (Miller et al. 2004).

In the first population-based birth cohort study to explore the relationship between ambient air pollution exposure and the risk of asthma incidence, we examined the effect of *in utero* and first-year exposures to ambient air pollutants, estimated at the individual level, on the risk of asthma diagnosis in children up to 3 and 4 years of age. Pollutant exposures investigated were carbon monoxide (CO), nitrogen oxides [nitric oxide (NO) and nitrogen dioxide (NO_2_)], particulate matter [≤ 10 μm and ≤ 2.5 μm in aerodynamic diameter (PM_10_ and PM_2.5_)], ozone (O_3_), sulfur dioxide (SO_2_), black carbon, woodsmoke, and proximity to roads and point sources.

## Materials and Methods

### Cohort identification

The cohort comprised all 1999 and 2000 births in southwestern British Columbia (BC) identified by linking administrative data sets from the BC Ministry of Health Services, the BC Vital Statistics Agency, and the BC Perinatal Database Registry (described by Brauer et al. 2008b). The study region includes the metropolitan centers of Vancouver (population 2,250,000) and Victoria (population 325,000) as well as the surrounding areas within the same airshed. To be eligible, children and their mothers had to be registered for the provincial medical plan (because registration is mandatory for provincial residents under a universal health care system, the entire resident population is effectively included) and reside in the study area for the duration of pregnancy and the first year of life. Children were excluded for low birth weight (< 2,500 g), preterm birth (< 37 weeks of gestation), or multiple births, given that these conditions are known strong risk factors for development of chronic respiratory conditions. In addition, these factors may confound the association between air pollution and asthma; low birth weight and gestational period have been found to be associated with both asthma and air pollution exposure in this cohort (Brauer et al. 2008b), as well as in other studies (Bobak 2000; Dik et al. 2004; Salam et al. 2005; Wang and Pinkerton 2007). Because low birth weight and gestational period may also act in the causal pathway between air pollution and lung effects, this exclusion may also bias the results to the null.

We used a nested case–control design to examine the association of air pollutants and incident asthma. Each asthma case was randomly matched to five controls from the birth cohort by sex and age (month and year of birth).

### Outcome measure

Asthma diagnoses among the cohort population were identified from physician billing records for primary care clinical encounters and from hospital discharge records, available to the end of 2003. The mean (± SD) age at end of follow-up was 48 ± 7 months and ranged from 36 to 59 months. Each contact with the health care system was recorded with a diagnostic code to indicate the primary reason for the contact. Asthma cases were defined as children with a minimum of two primary care physician diagnoses in a rolling 12-month period or a minimum of one hospital admission for asthma using the *International Classification of Diseases, 9th Revision* (WHO 1975) code 493 for asthma.

We also performed a sensitivity analysis to test the robustness of results to different administrative definitions of asthma. The analyses were repeated with *a*) a more inclusive definition of asthma that included children with only one asthma diagnosis (in hospital or primary care setting) and *b*) a more restrictive definition of asthma that included only children with at least three primary care asthma diagnoses or one hospital diagnosis.

### Covariates

The British Columbia Vital Statistics Agency (2009) database provided the birth date and sex of each child. In addition, we collected individual-level information on birth weight and gestational length (based on date of last menstrual period) from the BC Vital Statistics Clinical Birth Data (British Columbia Vital Statistics Agency 2009) and maternal smoking during pregnancy, maternal age, number of siblings, and intention to breast-feed from the BC Perinatal Database Registry (British Columbia Perinatal Health Program 2009), which collects this information on nearly all pregnancies in the province. First Nations (“status Indians”) status was obtained from hospital discharge records at birth. Individual-level data were not available on socioeconomic factors, so income quintiles and maternal education level quartiles were assigned at the level of Census dissemination areas (DAs) to approximate socioeconomic status. This is the smallest geographic area for which census data are distributed, with each DA containing approximately 400–700 people.

### Exposure measures

Air pollution exposure for each subject based on their residential address history was estimated using regulatory monitoring data, land use regression (LUR) modeling, and proximity to stationary pollution sources, with full details described elsewhere (Brauer et al. 2008a, 2008b; Henderson et al. 2007; Larson et al. 2007; Marshall et al. 2008). Exposures were assigned at the level of six-digit postal codes because full addresses were not available due to privacy laws. This corresponds to one edge of a block (block-face) in urban areas but is larger where population density is lower. For this cohort, 92% of the residential histories were georeferenced at the resolution of a city block or block-face. The regulatory monitoring network consists of 24-hr average measurements at 24 monitors for O_3_, 22 for NO and NO_2_, 14 for SO_2_, 19 for CO and PM_10_, and 7 for PM_2.5_. To assign exposures, the daily values at the three closest monitors within 50 km were weighted by their inverse distance (1/*d*) to the postal code of interest.

We used LUR modeling to develop high-resolution (10 m) traffic-related air pollution maps of NO, NO_2_, PM_2.5_, and black carbon (described by Brauer et al. 2008b; Henderson et al. 2007). Briefly, targeted intensive sampling campaigns measured nitrogen oxides, PM_2.5_, and particle absorbance (black carbon) in the region. Fifty-five geographic information system variables were available for predictive modeling, including road density, population density, elevation, and type of land use and were used to develop high-resolution (10 m) maps [for more information, see Brauer et al. 2008b; Henderson et al. 2007; see also Supplemental Material (doi:10.1289/ehp.0900916.S1 via http://dx.doi.org)]. These models have improved spatial resolution compared with the monitoring network approaches but lower temporal resolution. Cross-validation analysis showed that the models for NO and NO_2_ performed better than the models for PM_2.5_ and black carbon (Henderson et al. 2007).

We also used a targeted sampling campaign to develop a regional model of residential woodsmoke exposure (Larson et al. 2007). The spatial distribution of woodsmoke in the region was estimated through fixed-site and mobile monitoring of PM_2.5_ and levoglucosan (a marker for woodsmoke). These data, along with spatial covariates, were used to develop an LUR model to predict woodsmoke PM_2.5_ concentrations. Postal codes in the top tertile of exposure to woodsmoke PM_2.5_ were classified as being in a wood-burning area. Because woodsmoke is emitted seasonally, days were classified as wood-burning days based on a relationship between temperature (heating degree days) and measured concentrations of levoglucosan [for further details, see Brauer et al. 2008b; see also Supplemental Material (doi:10.1289/ehp.0900916.S1)]. Woodsmoke exposure was then estimated as the total number of burning days spent in a wood-burning area (divided by 10 for modeling).

We also used proximity to roadways and industrial point sources as estimates of ambient air pollution exposure. Residential postal codes were defined as being within 50 m or 150 m of highways and major roads (DMTI Canmap streetfile version 2006.3; DMTI Spatial Inc., Markham, Ontario, Canada). Proximity to roadway was treated as a dichotomous variable.

To estimate exposure to pollutants from industrial point sources (e.g., power plants, waste treatment facilities, paper production plants, and shipyards), each permitted point source was assigned an index value based on its pollutant contribution (PM_2.5_, sulfur oxides, nitrogen oxides, and volatile organic compounds) relative to other point sources in the region. Exposure at each postal code was then determined by an inverse distance–weighted (IDW) summation of emissions from point sources within 10 km (for further details, see Brauer et al. 2008b).

For all exposure metrics, an average exposure was calculated for the duration of pregnancy and the first year of life. Children were linked to the pollution maps using postal code(s) at the home residence(s). Residential histories were compiled using BC Ministry of Health data (2009), which includes records for in-patient hospital discharges, outpatient physician billing records, and medical plan registration. An individual’s residential postal code was recorded at each contact with the health care system. After excluding invalid and nonresidential postal codes (~ 10%), individual residential histories were constructed for mothers throughout pregnancy and children throughout the first year of life. An average exposure was calculated for gestation and the first year of life that was weighted by the time spent at each postal code.

The study methodology was reviewed and approved by the University of BC Behavioral Research Ethics Board (ethics certificate no. H07-01549).

### Statistical analysis

To assess the relationship of air pollution exposures on asthma diagnosis, we performed unadjusted and covariate-adjusted conditional logistic regression analyses. Covariates previously hypothesized to have an effect on asthma status (native status, breast-feeding, maternal smoking, income quintile, education quartile, maternal age, birth weight, and gestational length) were included in multivariate regressions based on statistical significance in bivariate regressions. Pollutants were entered into models in two ways: as continuous variables and as categorical variables based on the quartile of exposure. We calculated odds ratios (ORs) for continuous pollutants over a standard increase (1, 10, or 100 μg/m^3^) of the same order of magnitude as the interquartile range to allow for easy comparison with other studies. The models were repeated for each sex separately. Finally, for pollutants where *in utero* and first-year exposures were not too highly correlated (Pearson correlation coefficient < 0.70), we ran mutually adjusted models to help shed light on the relative importance of each exposure period.

## Results

### Study cohort description

BC Vital Statistics data identified 59,917 births in the region in 1999 and 2000. Of these, 41,565 (69.4%) children met the inclusion criteria of living in the study area during gestation and the first year of life and having complete medical plan registration through to 2003. We excluded 2,967 births because of low birth weight or preterm birth, 216 for multiple births, and 981 because of missing covariate information. Thus, we included 37,401 children (90%) in the final cohort from which we drew cases and controls. We excluded additional subjects for specific analyses where exposure information was not available.

A total of 3,482 children (9.3%) met the case definition for asthma and were included in the nested case–control analysis. Table 1 provides covariate information for the whole birth cohort, stratified by asthma status. Children meeting the case definition of asthma differed from the rest of the birth cohort for certain covariates: They tended to be born to mothers of younger age, lower education and income, and who were less likely to have initiated breast-feeding. Cases had slightly more siblings and tended to have lower birth weight and shorter gestational periods. We found no differences with respect to First Nations status or maternal reported smoking during pregnancy. Randomly picked controls were representative of the nonasthmatic children in the birth cohort as a whole.

### Exposure assessment

As expected for this study region (southwestern BC), the mean pollutant levels were low (Table 2) relative to international guidelines. The LUR model estimated higher exposures than the monitor-based assessments, in general, with the greatest discrepancy observed for NO. Because O_3_ was inversely correlated with the primary traffic-related pollutants (*r* = −0.7 to −0.9), the observed associations with O_3_ were largely protective. Also, the high O_3_ peaks that occur both daily and seasonally are lost through long-term averaging of exposure, further limiting our ability to examine the pollutant’s effects.

The correlations between different pollutants were generally high, and multipollutant models were not feasible.

Although many pregnancy and first-year exposures were highly correlated, NO, NO_2_, PM_10_, and black carbon had moderate correlations (ranging from 0.4 to 0.6) and could be examined together in mutually adjusted models [for full correlations, see Supplemental Material, Table 1 (doi:10.1289/ehp.0900916.S1)]. The overall trend was that average exposures were slightly lower in first year than in pregnancy with the exception of PM, NO, and SO_2_.

Asthmatic children had higher mean exposures for NO, NO_2_, CO, PM_10_, black carbon, SO_2_, and point sources (two-sided *t*-test *p* < 0.05) than did nonasthmatic children.

Table 2 also shows the number of children living in woodsmoke areas or in proximity to major roads (within 150 m from a highway or 50 m from a major road). Only a small number of children lived near major roads, and these did not significantly differ in asthma status (chi-square test *p* > 0.05). Residence in a woodsmoke area also did not differ by asthma status (chi-square test *p* > 0.05).

### Logistic regression

Table 3 provides the results of risk estimates for asthma diagnosis with our exposure measures *in utero* and during first year. The effect size estimates for first-year exposures are generally larger than for *in utero* exposures. Adjustment for covariates had relatively small effects on the resulting ORs, most often slightly increasing the effect estimates [unadjusted results available in Supplemental Material, Table 2 (doi:10.1289/ehp.0900916.S1)]. IDW exposure estimates for NO, NO_2_, CO, PM_10_, and SO_2_ all showed elevated risks of asthma diagnosis for both *in utero* and first-year average exposures. LUR modeling showed less consistent results. NO exposure was significantly associated with an elevated risk of asthma for *in utero* exposure and reached borderline statistical significance for first-year exposure. NO_2_ exposure was associated with an elevated risk of asthma for first-year exposures only. PM_2.5_ exposure was not associated with increased asthma risk for IDW or LUR exposure estimates. Black carbon exposure was associated with a 14% [95% confidence interval (CI), 1–29%] increase in asthma risk. An interquartile increase (30 points) on the industrial point source index was consistently associated with a 10–11% (95% CI, 4–18%) increase in asthma risk. Woodsmoke and proximity to roads were not associated with increased asthma risk.

Table 3 also provides the results for models including both the average *in utero* and first-year exposures for NO, NO_2_, PM_10_, and black carbon (and adjusted for covariates). Results for NO and PM_10_ show statistically significant ORs only for exposures occurring *in utero*, whereas for NO_2_ only the OR for first-year exposure is statistically significant. Black carbon shows similar (nearly statistically significant) point estimates for both periods.

**Figure 1:**
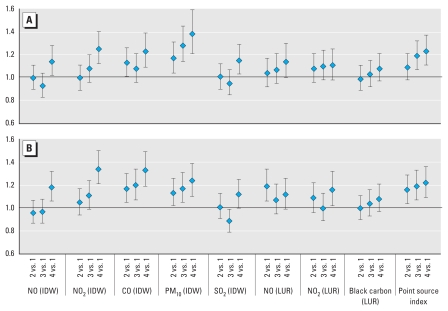
Results of conditional logistic regression by exposure quartile: average *in utero* (*A*) and first-year (*B*) exposure. Models are adjusted for parity, breast-feeding, income quintile (neighborhood level), maternal education status (neighborhood level), birth weight, and gestational length.

When we entered the pollutant exposures into models as categorical variables indicating the quartile of exposure, similar results emerged (Figure 1). Upper quartiles of exposure for NO, NO_2_, CO, PM_10_, SO_2_, black carbon, and point sources showed elevated ORs for asthma risk with respect to the lowest quartile of exposure. However, for many pollutants, including NO, NO_2_, CO, and SO_2_, the trend across quartiles was not consistently linear.

Regressions stratified by sex revealed that effect sizes were consistently larger for girls than for boys, with the exception of road proximity. As in the nonstratified analysis, PM_2.5_ and woodsmoke exposures were not associated with increased asthma risk for either sex. For the full results of sex-stratified analysis, see Supplemental Material, Table 3 (doi:10.1289/ehp.0900916.S1).

### Sensitivity analysis

We performed sensitivity analyses to test the robustness of the results to differing administrative data definitions of asthma. When we broadened the case definition to include children who had only one physician diagnosis of asthma, the resulting ORs for air pollution exposure were attenuated. Overall, we found a consistent trend that as the definition of asthma became more restrictive (three physician visits), associations with air pollution exposure strengthened. Restricting the asthma cases based on number of medical visits increases specificity of the definition and also likely indicates severity.

## Discussion

We found that higher exposure to ambient air pollution in early life was associated with elevated risks of asthma diagnosis in preschool-age children based on clinical records. Traffic-related pollutants (NO, NO_2_, CO, and black carbon) were associated with the highest risk estimates. PM_10_, SO_2_, and residence near industrial point sources were also associated with elevated asthma risk, whereas PM_2.5_, woodsmoke, and road proximity did not show elevated risks. The results from this population-based study strengthen the emerging evidence that air pollution exposure plays a role in childhood asthma development, although these findings should be confirmed in additional cohorts of children, particularly as they reach school age and asthma diagnosis is more robust.

The risk estimates we found were similar to the results of other birth cohort studies investigating respiratory outcomes. Morgenstern et al. (2008) also estimated air pollution exposure using LUR modeling and examined effects on risk of asthma diagnosis in 6-year-old children in Germany. They found ORs of 1.12 per 1-μg/m^3^ increase in PM_2.5_, 1.56 per 0.2 × 10^−5^/m increase in filter absorbance measure of black carbon, and 1.04 per 6.4-μg/m^3^ increase in NO_2_. Nordling et al. (2008) prospectively followed children in Stockholm, Sweden, from birth until 4 years of age and found that exposure to traffic-related air pollution during the first year of life was associated with an excess risk of persistent wheezing of 1.60 (95% CI, 1.09–2.36) for a 44-μg/m^3^ increase in traffic NO_x_. Brauer et al. (2007) used LUR models to estimate the effect of traffic-derived pollutants on asthma incidence among 4-year-old children in the Netherlands. They found ORs of 1.20 per 10-μg/m^3^ increase in NO_2_ and 1.30 per 0.6 × 10^−5^/m increase in filter absorbance (black carbon). They also found an elevated risk of 1.20 per 3.3-μg/m^3^ increase in PM_2.5_, which we did not identify in this study, although the composition of PM_2.5_ and the relationship between PM_2.5_ and other pollutants are likely to differ across locations. Compared with other studies/locations, PM_2.5_ in this study area was quite low and less variable. PM_2.5_ exposure estimates were also subject to more error because they were based on fewer monitors than other pollutants.

To our knowledge, this was the largest study and one of the few to examine the effects of *in utero* air pollution exposure on pediatric asthma risk. Mortimer et al. (2008) found that *in utero* exposure to air pollution was associated with negative effects on lung function in asthmatic children. Other effects of air pollution on the developing fetus include lower birth weight (Bobak 2000; Slama et al. 2007), small size for gestational age (Brauer et al. 2008b), preterm births (Bobak 2000; Brauer et al. 2008b; Ritz et al. 2000), and intrauterine mortality (Pereira et al. 1998).

Because of relatively high correlation between *in utero* and first-year exposures for many pollutants, we are unable to discern the relative importance of these exposure periods. Mutually adjusted models for NO, NO_2_, PM_10_, and black carbon did not consistently identify either period as more significant but did suggest that *in utero* exposures have an effect independent from postbirth exposures for NO and PM_10_. We cannot eliminate the possibility that these results were influenced by misclassification bias associated with a lack of temporal precision in the residential histories; *in utero* exposures were more likely to have been misclassified than first-year exposures because of greater confidence in residential postal code (which can be confirmed against the mother’s data after birth) and likely greater time spent at the residence after birth than during pregnancy. These exposure assessment errors would be expected to lead to a bias to the null for the intrauterine period. Further work is necessary to elucidate the relative importance of pre- and postbirth exposures.

Asthma risks due to air pollution were generally larger for girls for both *in utero* and first-year exposures. As expected, girls had a lower incidence of asthma (making up 36% of cases), but despite the smaller numbers, associations with pollutants were significantly elevated (except for woodsmoke, PM_2.5_, and road proximity). Several previous studies have also found that girls are more susceptible to air pollution, with higher risks of asthma (McConnell et al. 2006; van Vliet et al. 1997) and greater effects on lung function (Peters et al. 1999). However, this is not an entirely consistent finding (Gehring et al. 2002).

Proximity to roads is commonly used to approximate exposure because of its relative ease compared with monitoring methods. Although many studies have found asthma symptoms to be associated with proximity to major roads (Gauderman et al. 2005; McConnell et al. 2006; Morgenstern et al. 2007), this is also not a consistent finding (English et al. 1999). Simple proximity measures may not capture exposure accurately because they lack information on traffic density, vehicle mix, wind patterns, topography, land use characteristics, and other influences on pollution levels (Brauer et al. 2003; Henderson et al. 2007; Jerrett et al. 2005). This may explain why we did not find proximity to roads to be associated with increased asthma risk in this study. Our study was further challenged by the small number of children residing in proximity to major roads. Nonetheless, we did observe consistently elevated risks with measured and modeled values of traffic-derived pollutants, suggesting that traffic-related exposure is important.

The industrial point source index is subject to many of the same limitations as the road proximity measure. Despite these limitations, we observed elevated asthma risks associated with the point source index. This may partially indicate a socioeconomic effect; the point source index was among the only exposure indices that were reduced after adjustment for covariates, primarily as a result of adjustment for income and education status.

Residential woodsmoke contributes a considerable fraction of PM exposure in portions of the study area in the winter months (Ries et al. 2009). Despite this, woodsmoke exposure was not found to be associated with increased asthma risk. Previous studies have associated woodsmoke with adverse respiratory effects in children, including exacerbation of asthma (Allen et al. 2008; Zelikoff et al. 2002); however, its role in asthma development requires more research.

The use of linked administrative data sets presents some limitations, such as the lack of clinical details and information on asthma severity. However, our estimates of asthma incidence are consistent with previous findings in similar age ranges (Dik et al. 2004; Jaakkola et al. 2005). Furthermore, the validity of our findings is supported by a recent validation study of administrative data in a similar health care setting. It found that asthma codes were a highly sensitive and specific measurement of asthma in 0- to 5-year-olds compared with experts’ review of medical charts (To et al. 2006). Because of universal and free access to physician visits, we also believe that any misclassification of asthma status was nondifferential and therefore would be expected to bias the results to the null.

Limitations of the BC Perinatal Database Registry likely underlie the reason that we did not observe an expected effect of maternal smoking on asthma risk. The variable relies on maternal self-report and therefore likely includes some misclassified exposures due to a healthy reporting bias (e.g., Derauf et al. 2003).

An additional limitation of this study was the young age of the children. Wheezing illnesses in early childhood represent multiple phenotypes. Transient wheezing is common in infants and often resolves as the children age (Martinez et al. 1995; To et al. 2007). To et al. (2007) found that among children diagnosed with asthma before 6 years of age, 48.6% were in remission by 12 years of age. Children with a hospitalization for asthma or many physician visits for asthma were at greater risk of persistent asthma by 12 years of age (To et al. 2007). We have addressed this issue by restricting our asthma cases to children with a hospital admission or at least two outpatient diagnoses of asthma, because these indicate severe or ongoing symptoms, respectively. Sensitivity analyses requiring three outpatient diagnoses only made the resulting ORs larger, indicating that air pollution is associated with ongoing respiratory symptoms consistent with asthma. This indicates that adverse respiratory effects do occur with air pollution exposure, but to ensure associations with persistent asthma, the results must be confirmed when the children are older.

We were able to correct for a number of individual-level variables, but socioeconomic variables could be adjusted only at the neighborhood level. This is imperfect and may have led to some misclassification of socioeconomic status for individuals (Hanley and Morgan 2008); however, the adjustment generally had small, and often strengthening, effects on ORs. We also had no information on the child or family history of atopy, an important risk factor for asthma development and a potential effect modifier.

## Conclusion

In this population-based study, children with higher early life air pollution exposures, particularly to traffic-derived pollutants, were observed to have an increased risk of asthma diagnosis in the preschool years. This adds to evidence that outdoor air pollution not only exacerbates asthma but also may be associated with development of new disease. The risk increase is small at an individual level but presents a significant increase in burden of disease on a population level because in most urban and suburban settings, traffic-derived air pollution exposure is ubiquitous.

## Figures and Tables

**Table 1:** Covariate information for the children in the study [no. (%)].

Characteristic	Asthmatic children (*n* = 3,482)	Nonasthmatic children in total cohort (*n* = 33,919)	Randomly selected controls (*n* = 17,410)
Male sex*	2,222 (64)	16,885 (50)	11,110 (64)
Native status	70 (2)	528 (2)	282 (2)
Has siblings*	2,042 (59)	18,844 (56)	9,618 (55)
Breast-feeding initiation*	2,920 (84)	29,286 (86)	14,915 (86)
Maternal smoking status	268 (8)	2,625 (8)	1,400 (8)
Area-based income quintile^a^,*			
1	814 (23)	7,072 (21)	3,625 (21)
2	822 (24)	7,350 (22)	3,784 (22)
3	762 (22)	7,283 (21)	3,745 (22)
4	591 (17)	6,582 (19)	3,370 (19)
5	493 (14)	5,632 (17)	2,886 (17)
Area-based maternal education quartile^a^,*			
1	985 (28)	8,083 (24)	4,166 (24)
2	913 (26)	8,704 (26)	4,480 (26)
3	830 (24)	8,668 (26)	4,440 (26)
4	754 (22)	8,464 (25)	4,324 (25)
Maternal age (years)*	30.8 ± 5.2	31.0 ± 5.1	31.0 ± 5.1
Birth weight (g)*	3,510 ± 450	3,530 ± 460	3,539 ± 458
Gestational length (weeks)*	39.3 ± 1.2	39.4 ± 1.1	39.4 ± 1.1

Values are no. (%) or mean ± SD. Data are for children who met the case definition for asthma (two outpatient physician diagnoses within 12 months or one hospital diagnosis; first column), children in the total cohort who did not meet the asthma case definition (second column), and a randomly selected set of controls from the total cohort (five age- and sex-matched controls for each case; third column). The randomly selected controls were representative of the nonasthmatic cohort (with the exception of matched variables).

a Percentages may not add to 100 because of rounding error.

* Statistically significant difference (*p* < 0.05) between asthmatic and nonasthmatic children, chi-square test for frequencies and *t*-test for means.

**Table 2:** Mean exposure levels during pregnancy and the first year of life for preschool-age children with and without a diagnosis of asthma.

			*In utero*				First year			
				Percentile				Percentile		
Pollutant/method	Group	No.	Mean ± SD	25th	50th	75th	Mean ± SD	25th	50th	75th
NO/IDW (μg/m^3^)	Controls	16,970	19.82 ± 10.27	12.76	17.94	25.69	22.36 ± 10.39	15.76	20.25	27.98
	Asthma cases	3,394	20.32 ± 10.74	12.74	17.95	27.15	23.04 ± 10.88	15.84	20.56	30.25

NO/LUR (μg/m^3^)	Controls	14,005	30.38 ± 13.05	21.71	27.46	35.99	30.42 ± 12.13	23.09	27.05	34.28
	Asthma cases	2,801	31.03 ± 13.54	22.00	27.84	36.62	30.83 ± 12.67	23.39	27.11	34.39

NO_2_/IDW (μg/m^3^)	Controls	16,970	30.74 ± 8.90	24.96	31.50	36.54	29.86 ± 8.85	23.71	29.97	36.02
	Asthma cases	3,394	31.37 ± 9.20	25.17	32.15	37.57	30.68 ± 9.06	24.28	31.97	36.82

NO_2_/LUR (μg/m^3^)	Controls	14,005	31.68 ± 8.64	25.77	30.06	35.07	29.50 ± 5.29	25.90	28.73	32.70
	Asthma cases	2,801	31.73 ± 8.42	25.96	30.33	35.16	29.82 ± 5.46	26.16	28.87	33.08

CO/IDW (μg/m^3^)	Controls	16,240	612.2 ± 133.4	507.7	610.2	698.8	605.0 ± 135.2	512.9	607.2	690.1
	Asthma cases	3,248	618.8 ± 135.3	520.2	612.6	705.8	617.5 ± 132.5	521.6	614.5	707.8

O_3_/IDW (μg/m^3^)	Controls	16,795	30.48 ± 6.32	26.03	30.47	34.89	28.06 ± 4.86	25.09	28.33	31.57
	Asthma cases	3,359	30.05 ± 6.39	25.41	30.11	34.42	27.64 ± 4.94	24.32	28.11	31.28

PM_10_/IDW (μg/m^3^)	Controls	17,335	11.94 ± 1.35	11.08	12.15	12.85	12.37 ± 1.00	11.95	12.43	13.01
	Asthma cases	3,467	12.03 ± 1.30	11.21	12.23	12.89	12.42 ± 1.00	12.01	12.47	13.03

PM_2.5_/IDW (μg/m^3^)	Controls	16,775	4.74 ± 1.19	4.07	5.15	5.58	5.62 ± 0.61	5.35	5.60	6.13
	Asthma cases	3,355	4.71 ± 1.20	4.00	5.12	5.57	5.62 ± 0.61	5.35	5.62	6.13

PM_2.5_/LUR (μg/m^3^)	Controls	16,270	4.67 ± 2.47	3.08	4.25	5.98	4.50 ± 2.45	2.95	4.14	5.68
	Asthma cases	3,254	4.78 ± 2.46	3.194	4.33	6.02	4.59 ± 2.40	3.10	4.18	5.70

Black carbon^a^/LUR	Controls	16,270	1.34 ± 0.65	0.94	1.20	1.65	0.66 ± 0.33	0.46	0.70	0.89
	Asthma cases	3,254	1.37 ± 0.66	0.95	1.21	1.68	0.68 ± 0.33	0.49	0.72	0.91

SO_2_/IDW (μg/m^3^)	Controls	16,970	5.11 ± 2.40	3.70	4.48	6.20	5.22 ± 2.55	3.89	4.48	6.36
	Asthma cases	3,394	5.25 ± 2.51	3.69	4.49	6.74	5.37 ± 2.69	3.87	4.46	7.04

Point source index (*r* = 10 km)	Controls	17,410	23.69 ± 18.49	8.03	19.46	37.43	23.12 ± 18.71	7.63	18.29	37.03
	Asthma cases	3,482	25.18 ± 18.10	9.31	22.26	39.04	24.62 ± 18.36	8.91	20.60	38.42
		No.	Frequency	No. of exposure days (mean ± SD)			Frequency	No. of exposure days (mean ± SD)		

Woodsmoke	Controls	14,365	6,082 (42.3%)	60 ± 25			6,025 (41.9%)	89 ± 13		
	Asthma cases	2,873	1,269 (44.2%)	59 ± 25			1,256 (43.7%)	89 ± 13		

Proximity to major roads^b^	Controls	17,410	859 (4.9%)	—			658 (3.8%)	—		
	Asthma cases	3,482	181 (5.2%)	—			135 (3.9%)	—		

a Unit = 10^−5^/m increase in filter absorbance.

b Number of children with residence < 150 m from a major road or < 50 m from a highway.

**Table 3:** Adjusted ORs for asthma risk due to average exposures during pregnancy and the first year of life for LUR and IDW exposure metrics.

Pollutant/method	Exposure interval	*In utero* exposure (95% CI)	First-year exposure (95% CI)
NO/IDW	10 μg/m^3^	1.07 (1.03–1.12)	1.08 (1.04–1.12)
NO/LUR	10 μg/m^3^	1.05 (1.02–1.09)	1.03 (1.00–1.07)
NO_2_/IDW	10 μg/m^3^	1.10 (1.05–1.15)	1.12 (1.07–1.17)
NO_2_/LUR	10 μg/m^3^	1.02 (0.97–1.07)	1.13 (1.04–1.23)
CO/IDW	100 μg/m^3^	1.07 (1.04–1.10)	1.10 (1.06–1.13)
O_3_/IDW	10 μg/m^3^	0.83 (0.77–0.89)	0.81 (0.74–0.87)
PM_10_/IDW	1 μg/m^3^	1.09 (1.05–1.13)	1.07 (1.03–1.12)
PM_2.5_/IDW	1 μg/m^3^	0.95 (0.91–1.00)	1.05 (0.97–1.14)
PM_2.5_/LUR	1 μg/m^3^	1.02 (1.00–1.03)	1.01 (0.99–1.03)
Black carbon/LUR	10^−5^/m increase in filter absorbance	1.08 (1.02–1.15)	1.14 (1.01–1.29)
SO_2_/IDW	1 μg/m^3^	1.03 (1.02–1.05)	1.03 (1.02–1.05)
Point source index (*r* = 10 km)	30 points	1.11 (1.04–1.18)	1.10 (1.04–1.17)
Woodsmoke	10 burn days	1.00 (0.98–1.01)	1.00 (0.99–1.01)
Road proximity	0/1	0.97 (0.82–1.15)	1.01 (0.84–1.22)
Mutually adjusted for *in utero* and first-year exposures			
NO/LUR	10 μg/m^3^	1.06 (1.01–1.11)	0.99 (0.95–1.04)
NO_2_/LUR	10 μg/m^3^	0.98 (0.92–1.03)	1.15 (1.05–1.27)
PM_10_/IDW	1 μg/m^3^	1.10 (1.04–1.15)	0.99 (0.93–1.5)
Black carbon/LUR	10^−5^/m increase in filter absorbance	1.06 (1.00–1.14)	1.09 (0.96–1.24)

All models are adjusted for parity, breast-feeding, income quintile (neighborhood level), maternal education status (neighborhood level), birth weight, and gestational length. Additionally, results of models that include both *in utero* and first-year exposures are provided for NO, NO_2_, PM_10_, and black carbon (with Pearson correlation coefficients < 0.70).
